# African Swine Fever Virus Induces STAT1 and STAT2 Degradation to Counteract IFN-I Signaling

**DOI:** 10.3389/fmicb.2021.722952

**Published:** 2021-08-26

**Authors:** Elena Riera, Daniel Pérez-Núñez, Raquel García-Belmonte, Lisa Miorin, Adolfo García-Sastre, Yolanda Revilla

**Affiliations:** ^1^Microbes in Health and Welfare Department, Centro de Biología Molecular Severo Ochoa, CSIC-UAM, c/Nicolás Cabrera, Madrid, Spain; ^2^Department of Microbiology, Icahn School of Medicine at Mount Sinai, New York, NY, United States; ^3^Icahn School of Medicine at Mount Sinai, Global Health and Emergent Pathogens Institute, New York, NY, United States; ^4^Division of Infectious Diseases, Department of Medicine, Icahn School of Medicine at Mount Sinai, New York, NY, United States; ^5^Icahn School of Medicine at Mount Sinai, The Tisch Cancer Institute, New York, NY, United States; ^6^Department of Pathology, Molecular and Cell-Based Medicine, Icahn School of Medicine at Mount Sinai, New York, NY, United States

**Keywords:** ASFV, Arm/07/CBM/c2, NH/P68, JAK/STAT, IFN-I pathway, STAT1, STAT2, virulence

## Abstract

African swine fever virus (ASFV) causes a serious disease in domestic pigs and wild boars and is currently expanding worldwide. No safe and efficacious vaccines against ASFV are available, which threats the swine industry worldwide. African swine fever virus (ASFV) is a complex dsDNA virus that displays multiple mechanisms to counteract the host innate immune response, whose efficacy might determine the different degrees of virulence displayed by attenuated and virulent ASFV strains. Here we report that infection with both virulent Arm/07/CBM/c2 and attenuated NH/P68 strains prevents interferon-stimulated gene (ISG) expression in interferon (IFN)-treated cells by counteracting the JAK/STAT pathway. This inhibition results in an impaired nuclear translocation of the interferon-stimulated gene factor 3 (ISGF3) complex, as well as in the proteasome-dependent STAT2 degradation and caspase 3-dependent STAT1 cleavage. The existence of two independent mechanisms of control of the JAK/STAT pathway, suggests the importance of preventing this pathway for successful viral replication. As ASFV virulence is likely associated with the efficacy of the IFN signaling inhibitory mechanisms, a better understanding of these IFN antagonistic properties may lead to new strategies to control this devastating pig disease.

## Introduction

African swine fever virus (ASFV) is the causative agent of African swine fever (ASF) disease, which affects domestic pigs and wild boars (Viñuela, 1985). Depending on the virulence of the viral strain, disease manifestations include chronical or subclinical forms as well as acute hemorrhagic forms (Gómez-Villamandos et al., 2013) that can result in death of the infected hosts in few days (Blome et al., 2013). Despite the disease was eradicated from Europe in 1995, the 2007 outbreak in the Caucasus resulted in the spreading of the virus to neighboring countries including Russia and more recently also Bulgaria, Belgium and Germany, with more than 500 outbreaks reported up to date in Europe. Importantly, since 2018 multiple ASFV outbreaks have also been reported across Asia reaching 190 outbreaks in China (Situational updates of ASF in Asia and the Pacific - OIE - Asia, n.d.), putting the pig industry at serious risk due to the lack of an available efficient and safe vaccine.

The high complexity of ASFV (Viñuela, 1985), a large enveloped nucleocytoplasmic dsDNA virus (García-Beato et al., 1992; Rojo et al., 1999; Ballester et al., 2010), lies on its long genome of 170–190 kbs, which encodes for more than 150 proteins with specific roles in various stages of the viral life cycle (Yáñez et al., 1995; Nogal et al., 2001; Carrascosa et al., 2002; Hernáez et al., 2004; Granja et al., 2004, 2006a,b, 2008, 2009; Hurtado et al., 2011; Dixon et al., 2013; Sánchez et al., 2013; Pérez-Núñez et al., 2015; Banjara et al., 2017; Quintas et al., 2017). The tropism of the virus is generally restricted to cells of the monocyte-macrophage lineage (Viñuela, 1985; Gómez-Villamandos et al., 2013). These cells are crucial players in the host defense against infection and constitute one of the key effectors of the innate immune response. Therefore, the establishment of successful innate immunity evasion strategies may be required to initiate a productive infection.

Type I interferon, or IFN-I, is a critical mediator in the host’s innate immune response. IFN-I is rapidly induced after microbial sensing by pattern recognition receptors (PRRs), and secreted by the infected cells (Kawai and Akira, 2006; Thompson et al., 2011). Once secreted, IFN-I acts both in autocrine and paracrine manners via the activation of the JAK/STAT signaling pathway. IFN-I binds to its receptor (IFNAR, composed by IFNAR1 and IFNAR2 subunits) to trigger the activation of the receptor-associated kinases JAK1 and TYK2 (Remy et al., 1999), which in turn regulate the phosphorylation of STAT1 and STAT2 and the formation of the ISGF3 complex. The ISGF3 complex, composed of phosphorylated STAT1 and STAT2, and the IFN regulatory factor 9 (IRF9), translocates into the nucleus and triggers the expression of many interferon-stimulated genes (ISGs) and the establishment of an antiviral state (Darnell et al., 1994; Meyer and Vinkemeier, 2004; Stark and Darnell, 2012). Several viruses, such as adenoviruses (Leonard and Sen, 1996) and flaviviruses (Ashour et al., 2009; Grant et al., 2016) have been shown to degrade STAT1, STAT2 and/or IRF9, to escape this antiviral response. In addition, the translocation of the ISGF3 complex into the nucleus has also been reported to be counteracted by viruses, such as Ebolavirus (EV) (Reid et al., 2006), Porcine Reproductive and Respiratory Syndrome virus (PRRSV) (Patel et al., 2010; Wang et al., 2013), enterovirus (Wang et al., 2017) or SARS-CoV-2 (Miorin et al., 2020).

Previous studies have shown that virulent strains of ASFV are able to modulate IFN-I induction (Gil et al., 2003, 2008; Correia et al., 2013; Fishbourne et al., 2013; Reis et al., 2016). In this regard, we recently reported that the virulent Arm/07 ASFV strain, but not the attenuated NH/P68 strain, is able to counteract the cGAS-STING pathway to efficiently impair IFN-β production (García-Belmonte et al., 2019). Here we show, for the first time, that both the virulent Arm/07/CBM/c2 (Gallardo et al., 2015; Pérez-Núñez et al., 2020) and the attenuated NH/P68 (Leitão et al., 2001) ASFV strains can counteract the JAK/STAT IFN signaling pathway in porcine alveolar macrophages (PAM) at multiple levels. We have identified two different strategies employed by ASFV to inhibit IFN-I signaling in PAMs. On one hand, we discovered that both virulent Arm/07/CBM/c2 and attenuated NH/P68 strains can trigger proteasome-dependent degradation of STAT2. On the other hand, we also found that infection with both strains results in caspase-3-dependent STAT1 cleavage. Caspase-3 activation during ASFV infection is consistent with previous studies that have found an association between apoptosis and ASFV replication *in vitro* (Nogal et al., 2001) and *in vivo* (Ramiro-Ibáñez et al., 1996; Oura et al., 1998; Gómez-Villamandos et al., 2013). Moreover, we have previously described that apoptosis is induced at early steps of ASFV infection (Carrascosa et al., 2002). Interestingly, both STAT1 and STAT2 degradation are less pronounced during NH/P68 infection, which could help to explain at least in part the low virulence pattern observed in pigs after infection with this attenuated ASFV strain (Gallardo et al., 2018).

## Materials and Methods

### Cells and Viruses

Porcine alveolar macrophages (PAM) were obtained by bronchoalveolar lavage as previously described (Carrascosa et al., 1982) and were cultured in Dulbecco modified Eagle medium (DMEM) supplemented with 2 mM L-glutamine, 0.4 mM non-essential amino acids and 100 U/ml gentamicin with 10% porcine serum. COS-1 cells from African green monkey kidney were obtained from the American Type Culture Collection (ATCC) and grown in DMEM supplemented with 2 mM L-glutamine, 0.4 mM non-essential amino acids, 100 U/ml gentamicin and 5% fetal bovine serum (FBS) (Invitrogen Life Technologies). The field attenuated ASFV strain NH/P68 (non-hemoabsorbing virus Portugal 68) (Leitão et al., 2001) and the field virulent strain Armenia/07/CBM/c2 (Gallardo et al., 2015; Pérez-Núñez et al., 2020) were propagated in PAM and titrated by plaque assay on COS-1 cells or by hemadsorption with erythrocytes in PAM, respectively, as previously described (Carrascosa et al., 1982; García-Belmonte et al., 2019; Pérez-Núñez et al., 2020).

### Antibodies and Reagents

Monoclonal rabbit antibodies anti-pSTAT1 (Tyr701, D4A7), anti-pSTAT2 (Tyr690, D3P2P), anti-STAT1 (D1K9Y), anti-STAT2 (D9J7L), polyclonal rabbit antibody anti-ISG15 (2743) and anti-ubiquitin (3933) were acquired from Cell Signaling. Monoclonal mouse antibody anti-β-actin (C-4, sc-47778) and the secondary antibody anti-m-IgGκ (BP-HRP, sc-516102) were purchased from Santa Cruz Biotechnology. Monoclonal antibodies anti-p32 ASFV protein (S-1D8 and S-5C1) were kindly provided by S.Y. Sunwoo. Rabbit antibody anti-p17 ASFV protein was previously generated in our lab (Suárez et al., 2010). Antiserum 1262 was kindly provided by E. Tabarés. For Western blot experiments, anti-rabbit and anti-mouse immunoglobulin G coupled to peroxidase antibodies and ECL Prime Western blotting detection reagent were purchased from Amersham Biosciences. Secondary antibodies anti-rabbit/Alexa Fluor 488 and anti-mouse/Alexa Fluor 555 from Invitrogen were used in Immunofluorescence.

Proteasome inhibitor MG132, lysosome/autophagosome inhibitor chloroquine (CQ) or caspase-3 inhibitor Ac-DEVD-CMK (Sigma-Aldrich) were used at a 20, 50, or 80 μM concentration, respectively, during infection. For dose dependent experiments, the concentrations used were: 1, 5, or 20 μM for MG132 inhibitor and 10, 40, or 100 μM for Ac-DEVD-CMK inhibitor as indicated. For the analysis of STAT1 and STAT2 expression levels, cytosine arabinoside (AraC) was employed at a concentration of 40 μg/ml during infection. For STAT1/2 phosphorylation experiments, ISG and ISRE induction, cells were treated with universal type I IFN at the indicated concentrations (PBL; catalog no 11200-1).

### Luciferase Reporter Assays

COS-1 cells seeded in M24 plates were co-transfected with pISRE-firefly-luc (50 ng/well) and Renilla luciferase reporter construct pRLTK (25 ng/well). At 24 h post-transfection, cells were mock infected or infected with NH/P68 or Arm/07/CBM/c2 isolates (1 PFU/cell) in DMEM 2% FBS. At 1 h post-infection, cells were either mock treated or treated with 1,000 U/ml of universal type I IFN (PBL). At 16 hpi, cells were collected and processed according to the manufacturer indications and the luciferase activity was measured using the Luc-Pair^TM^ Duo-Luciferase HS Assay Kit (GeneCopoeia) and carried out with FLUOstar OPTIMA reader (BMG LabTech).

### RNA Extraction and RT-qPCR

6 × 10^6^ PAM were seeded in p60 plates and mock infected or infected with either Arm/07/CBM/c2 or NH/P68 strains (1 PFU/cell) in DMEM 10% porcine serum. At the indicated times post-infection, cells were either mock treated or treated with 1,000 U/ml of universal type I IFN (PBL) for 8 h. The total mRNA was extracted using the RNeasy kit (Qiagen) and cDNA was obtained using NZY first-strand cDNA synthesis kit (NZYTech). STAT1, STAT2, ISG15, MxA, p32, and p72 mRNA levels were evaluated by real-time PCRs using ABI PRISM 7900HT Fast (ThermoFisher) detection system. Gene expression levels were normalized to the housekeeping gene 18S rRNA and these values where then relativized to the mock infected and untreated sample. The primers used were: 5′-AGCAAGCGTAACCTTCAGGA-3′ and 5′-TGAATCTCTGG GCATTTTCC-3′ for STAT1 detection; 5′-GCAGGAAAGGG CAACAATAA-3′ and 5′-GAGGGTGTCCGTTGTCAGTT-3′ for STAT2 detection; 5′-GGTGCAAAGCTTCAGAGACC-3′ and 5′-GTCAGCCAGACCTCATAGGC-3′ for ISG15 detection; 5-A GAAGACGAATGGAAGGGCA-3′ and 5′-GACTTCCTTTTCC ACCTGCG-3′ for MxA detection; 5′-AAAAATGATAATG AAACCAATGAATG-3′ and 5′-ATGAGGGCTCTTGCTCAAA C-3′ for viral p32 detection; 5′-TGCATAGGAGAGGGCCACTA-3′ and 5′-CCAGGGGATAAAATGACTGG-3′ for viral p72 detection and 5′-GGCCCGAGGTTATCTAGAGTC-3′ and 5′ -TCAAAACCAACCCGGTCA-3′ for porcine 18S detection.

### Western Blots

PAM were seeded, mock infected or infected with ASFV as indicated and treated or not with 1,000 U/ml of universal type I IFN for 1 h. At the indicated times post-infection cells were harvested, washed with PBS, and lysed with radioimmunoprecipitation assay (RIPA) buffer (50 mM Tris-HCl pH 7.4, 150 mM NaCl, 1% Triton, 0.5% Deoxycholate, SDS 0.1%) supplemented with protease and phosphatase cocktail inhibitors (Roche). Lysates were centrifuged at 13,000 rpm for 10 min at 4°C and the supernatants were collected. Protein concentration was determined using a Pierce BCA Protein Assay kit (ThermoScientific). Equal protein amounts were resolved by sodium dodecyl sulfate polyacrylamide gel electrophoresis (SDS-PAGE) and transferred to Immobilon-P membrane (Millipore). Membranes were incubated with the following antibodies: anti-STAT1 (1: 2,000), anti-STAT2 (1: 1,000), anti-pSTAT1 (1: 1,000), anti-pSTAT2 (1: 500), anti-ISG15 (1: 1,000), anti-ubiquitin (1: 1,000), anti-β-actin (1: 6,000), anti-p17 (1: 1,000), and anti-p32 (S-1D8, 1: 6,000). Porcine serum 1262 was used to detect viral p72 protein (1: 1,000). After three wash steps, membranes were exposed to specific peroxidase-conjugated secondary antibodies: anti-mouse (1: 2,000), anti-rabbit (1: 5,000), anti-pig (1: 7,000) or anti-m-IgGκ secondary antibody (1: 1,000) immunoglobulin G coupled to peroxidase. Protein bands were visualized by chemiluminescence detection using ECL Prime (Amersham Biosciences).

### Cellular Fractionation

PAM were seeded in p60 plates (6 × 10^6^ cells/plate) and mock infected or infected with NH/P68 or Arm/07/CBM/c2 (2 PFU/cell) for 7 or 15 h. Cells were then mock treated or treated with IFN-I (250 U/ml) for 1 h. The whole cell extract (WCE), cytoplasmic fraction and chromatin fraction were isolated as described previously (Méndez and Stillman, 2000; García-Belmonte et al., 2019). Briefly, cells were resuspended in a buffer composed by 10 mM HEPES (pH 7.9), 10 mM KCl, 1.5 mM MgCl2, 0.34 M sucrose, 10% glycerol, 1 mM dithiothreitol (DTT), 0.1 mM phenylmethylsulfonyl fluoride (PMSF), protease and phosphatase inhibitors (Roche). This lysate corresponded to the WCE. Then, Triton X-100 was added followed by incubation for 5 min on ice. The nuclei were then centrifuged for 4 min at 3,600 rpm at 4°C. The supernatant was collected and centrifuged at 14,000 rpm for 15 min at 4°C corresponding to the cytoplasmic fraction. The pellet obtained in the first centrifugation was washed with the previous buffer and resuspended in a buffer containing 3 mM EDTA, 0.2 mM EGTA, 1 mM DTT, 0.1 mM PMSF and protease and phosphatase inhibitors (Roche). After incubation on ice for 30 min, the pellet lysate was centrifuged for 4 min at 4,000 rpm at 4°C. The pellet corresponding to the chromatin fraction was washed and resuspended in loading buffer.

### Immunofluorescence

PAM were grown on coverslips, mock infected or infected and mock treated or treated with 250 U/ml of universal type I IFN at 7 or 15 hpi for 1 h. Then, cells were fixed with 4% paraformaldehyde for 20 min at room temperature (RT) and 10 min with methanol at −20°C. Cells were blocked with 5% porcine serum and 0.3% Triton^TM^ X-100 in PBS for 1 h. Cells were then stained with the primary antibodies anti-phospho-STAT1 (1: 50), anti-phospho-STAT2 (1: 50), anti-STAT1 (1: 250), anti-STAT2 (1: 150) and anti-p32 (1: 200) diluted in PBS 1% BSA 0.3% Triton X-100 overnight at 4°C. Cells were washed twice with PBS and incubated with the fluorescent-conjugated secondary antibodies anti-rabbit/Alexa Fluor 488 (1: 500) or anti-mouse/Alexa Fluor 555 (1: 500) diluted in 1% BSA, 0.3% Triton X-100 in PBS for 1 h. Cells were washed twice with PBS and then the coverslips were mounted with DAPI Fluoromount-G (SouthernBiotech). Images were taken by using Nikon A1R + *in vivo* coupled to an inverted Eclipse Ti-E microscope (Nikon) with a 60x oil immersion objective lens. Images were imported into Image J software for analysis. To calculate the nuclear mean fluorescence of pSTAT1, pSTAT2 or IRF9, first, the nuclear surface was delimited by using the Li Threshold Image J tool, taking the DAPI staining as reference. “Fill holes” tool was used to cover the entire nuclear surface and was manually checked prior to analysis. Finally, the mean fluorescence within the delimited nuclei corresponding to IRF9, pSTAT1 or pSTAT2 was calculated. More than 70 cells per condition was averaged from three independent replicates.

### Immunoprecipitation Assay

8 × 10^6^ COS-1 cells were co-transfected with 3 μg of pCAGGS empty vector (chicken β-actin promoter) or with human-STAT2-FLAG (Miorin et al., 2019) and pCI-His-hUbiquitin vector (Addgene, #31815). At 6 h post-transfection, cells were mock-infected or infected with Arm/07/CBM/c2 strain. At 16 hpi, cells were collected and lysed following the Pierce^TM^ Classic Magnetic IP/Co-IP Kit (ThermoFisher; catalog code 88804) manufacturer instructions. A portion of the lysates was used as “Input” and the rest was employed to the immunoprecipitation (IP). A/G magnetics beads were washed and bound to STAT2 antibody (1: 50 dilution). A crosslink of the beads attached to the antibody was made to promote an irreversible binding, following the manufacturer instructions. Subsequently, the rest of the lysate was incubated with the beads bound to the antibody overnight at 4°C under rolling agitation. Beads were then washed and the bound protein eluted with Kit’s elution buffer. Input and immunoprecipitated samples were loaded into SDS-PAGE followed by immunoblot analysis. Antibodies against ubiquitin, STAT2 and actin were employed.

## Results

### Attenuated NH/P68 and Virulent Arm/07/CBM/c2 ASFV Infection Suppresses Type I IFN-Mediated Signaling

**FIGURE 1:**
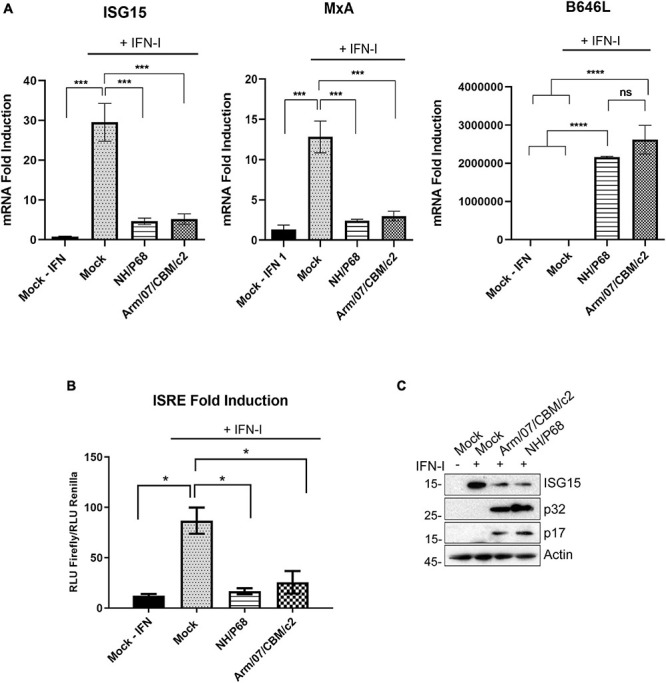
NH/P68 and Arm/07/CBM/c2 ASFV strains are able to impair IFN-I signaling. PAMs were either mock infected or infected with NH/P68 or Arm/07/CBM/c2 ASFV strains (1 PFU/cell). At 16 h post-infection, cells were mock treated or treated with universal type I IFN (1,000 U/ml) for 8 h. ISG15 and MxA mRNAs were measured by qRT-PCR **(A)**. As a control of infection, ASFV viral B646L gene mRNA was determined. The obtained values were relativized against the mock infected and untreated sample. All data are means ± SEM (*n* = 3). One-way ANOVA statistical analysis and Tukey’s multiple comparison test were performed (*p < 0.05, ***p < 0,001; ****p < 0,0001). ISRE-luciferase reporter assay in mock infected or infected COS-1 cells with either NH/P68 or Arm/07/CBM/c2 strains (1 PFU/cell, 16hpi) treated or not with type I IFN (1,000 U/ml) **(B).** Graphs represent the means of the Firefly luciferase RLU (Relative Luminiscence Units) values divided by its Renilla luciferase RLU values from biological triplicates. The obtained values were relativized against the mock infected and untreated sample. Two-tailed *t*-test comparison made with Mock + IFN (*p < 0.05). Western blot analysis of ISG15, viral p17, p32 and actin expression in COS-1 cells lysates previously used for the luciferase assay **(C)**.

We previously described that the virulent ASFV Arm/07 strain, but not the attenuated NH/P68 strain, inhibits IFN-β production by blocking the cGAS-STING pathway in PAM (García-Belmonte et al., 2019). In this study, we planned to gain further insights into the control of innate immune response by attenuated and virulent ASFV strains by assessing the ability to suppress the IFN-I signaling pathway. To achieve this, we analyzed the induction of IFN-I stimulated genes (ISGs) by qRT-PCR in control or infected cells that were either mock treated or treated with IFN-I (1,000 U/ml). As shown in Figure 1A, ISG15 and MxA expression levels in NH/P68 or Arm/07/CBM/c2 infected PAMs were 5-fold lower than those detected in mock infected cells after IFN-I treatment. Expression of the viral B646L gene (coding for p72 ASFV protein) was measured as control and showed equivalent expression levels in cells infected with both strains.

To further demonstrate that NH/P68 and Arm/07/CBM/c2 viruses are able to counteract the IFN-I signaling pathway, we also assessed the IFN-dependent activity of the interferon-stimulated response element (ISRE) promoter in mock infected or ASFV infected COS-1 cells by performing a Dual-Luciferase reporter gene assay (Figures 1B,C). In agreement with our qRT-PCR data, infection with either NH/P68 or Arm/07/CBM/c2 strains strongly inhibited the IFN-I-dependent ISRE activation (Figure 1B). In addition, the expression of endogenous ISG15 was also efficiently suppressed by infection with both NH/P68 and Arm/07/CBM/c2 ASFV strains in COS-1 cells treated with IFN-I (Figure 1C), both the unconjugated (15 kDa) and a conjugated (35 kDa) form of ISG15 (Supplementary Figure 1). Altogether, these results suggest that both NH/P68 and Arm/07/CBM/c2 strains are able to interfere with the activation of the JAK/STAT signaling pathway.

### ASFV NH/P68 and Arm/07/CBM/c2 Prevent IRF9/STAT1/STAT2 Nuclear Translocation and Impair IFN-I-Induced STAT1 and STAT2 Phosphorylation

**FIGURE 2:**
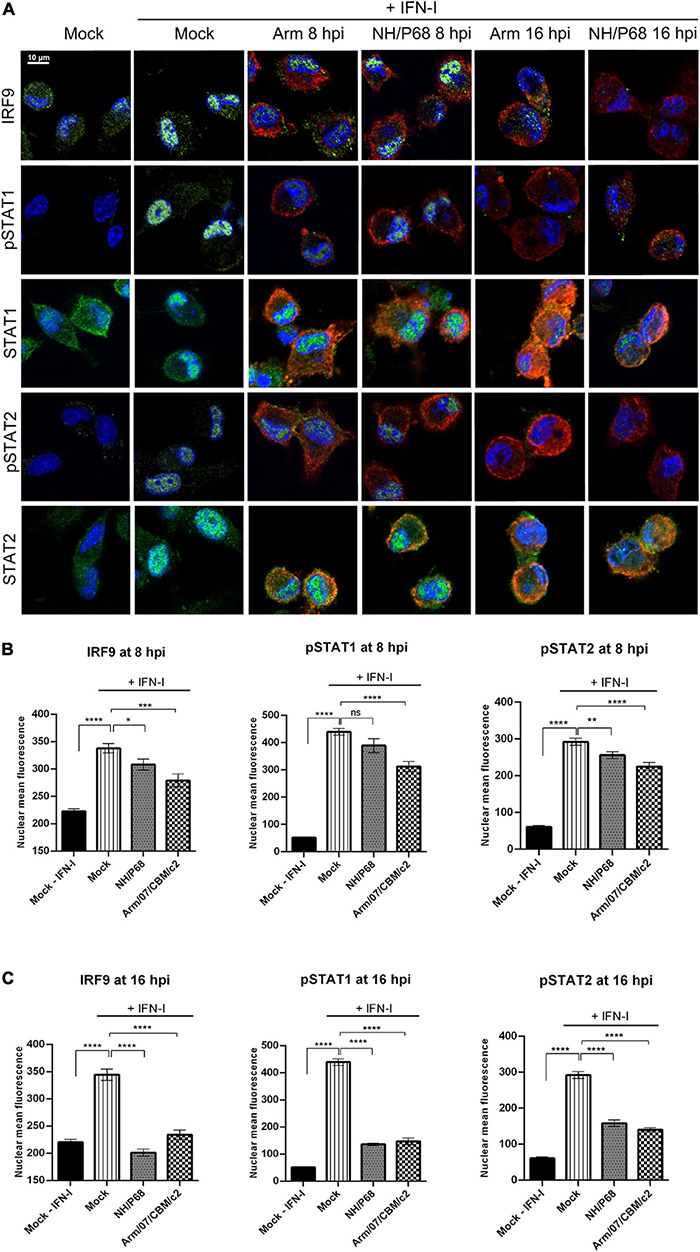
NH/P68 and Arm/07/CBM/c2 strains interfere with ISGF3 nuclear translocation and inhibits STAT1 and STAT2 phosphorylation at late times of infection. PAMs were mock infected or infected with NH/P68 or Armenia/07/CBM/c2 (Arm) (1 PFU/cell). At 7 or 15 hpi, cells were untreated or treated with universal type I IFN (250 U/ml). After 1 h of treatment, cells were fixed and stained with DAPI (blue), anti-IRF9/anti-pSTAT1/anti-STAT1/anti-pSTAT2/anti-STAT2 (green) and anti-p32 (red) antibodies and examined by a confocal microscope. Merged images of the different channels are shown **(A)**. The nuclear mean fluorescence of mock infected cells or infected cells (p32 labeled) at 8 hpi **(B)** or at 16 hpi **(C)** was measured using Image J software. The nuclear mean fluorescence of IRF9, pSTAT1 or pSTAT2 was calculated among 70–100 cells per condition of three biological replicates. All data are means ± SEM. Data were statistically analyzed by using a Student *t* test (*, *P* < 0.05; **, *P* < 0.01; ***, *P* < 0. 001; ****, *P* < 0.0001).

In order to elucidate the molecular mechanism employed by ASFV to counteract the activation of the JAK/STAT signaling pathway, we then explored the intracellular localization of the ISGF3 complex factors in mock infected or infected cells upon treatment with recombinant IFN-I. IRF9, together with phosphorylated STAT1 and STAT2, forms the ISGF3 complex which translocates into the nucleus upon IFN-I induction to mediate ISGs transcription. For this aim, PAM cells were mock infected or infected with either NH/P68 or Arm/07/CBM/c2 (1 PFU/cell), and then mock treated or treated with IFN-I (250 U/ml) for 1 h before fixation. As expected, confocal microscopy analysis revealed that endogenous IRF9, STAT1 and STAT2 were mostly localized in the cytoplasm in mock infected cells not treated with IFN-I, while a nuclear localization was observed in IFN-I-treated control cells (Figure 2A and Supplementary Figure 2). Interestingly, while IRF9, STAT1, and STAT2 were found in the nucleus of IFN-I treated PAM cells after 8 hpi, their localization was primarily cytoplasmic after 16 hpi, suggesting that both attenuated and virulent strains impair the nuclear translocation at late times of the infection. Given these results, we then assessed the phosphorylation of STAT1 and STAT2 as well as their intracellular localization in response to IFN-I treatment in mock infected vs. infected cells (Figure 2A and Supplementary Figure 3). As expected, STAT1 and STAT2 phosphorylation and nuclear translocation were clearly detected in mock infected cells that were treated with IFN-I for 1 h. However, cells infected with NH/P68 or Arm/07/CBM/c2 before IFN-I treatment exhibited a decrease in the levels of nuclear pSTAT1 and pSTAT2 that was particularly evident at 16 hpi. Accordingly, the quantification of nuclear IRF9, pSTAT1, and pSTAT2 during infection with both virulent and attenuated ASFV stains showed a slight but significant decrease in cells treated at 8 hpi (Figure 2B), and a more dramatic drop at 16 hpi (Figure 2C).

**FIGURE 3:**
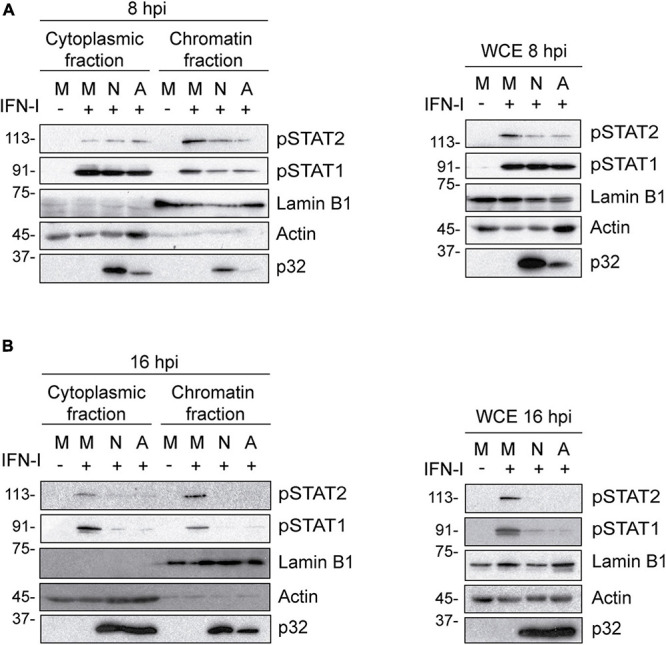
The IFN-I-dependent nuclear localization of pSTAT1 and pSTAT2 is impaired during NH/P68 and Arm/07/CBM/c2 infection. PAMs were mock infected (M) or infected with NH/P68 (N) or Armenia/07/CBM/c2 (A) strain (2 PFU/cell) for 8 **(A)** or 16 **(B)** hours. At 7 or 15 hpi, cells were untreated or treated with type I IFN (250 U/ml) during 1 h. Then, cells were collected and nuclear fractionation was performed. The whole cell extract (WCE), cytoplasmic fraction and nuclear chromatin fraction were analyzed by 10% SDS-PAGE, followed by immunoblotting with anti-pSTAT2, anti-pSTAT1 and anti-p32 (viral early protein) antibodies. As controls of the fractionation, nuclear antibody against lamin B1 and cytoplasmic antibody against actin were used.

Nucleus-cytoplasm fractionation experiments further supported our findings. As shown in Figure 3A, while the levels of pSTAT1 and pSTAT2 in the cytoplasmic fraction were similar in mock and infected cells, their levels decreased in the chromatin fraction of cells infected with either NH/P68 or Arm/07/CBM/c2 for 8 h. Remarkably, pSTAT1 and pSTAT2 levels were strongly reduced in both fractions of IFN-I treated cells at 16 hpi (Figure 3B). Altogether, these data indicate that both attenuated NH/P68 and virulent Arm/07/CBM/c2 strains significantly impair the phosphorylation of STAT1 and STAT2, and the nuclear translocation of the ISGF3 complex to overcome the IFN-mediated antiviral response.

### ASFV Attenuated and Virulent Strains Modulate STAT1 and STAT2 Levels

**FIGURE 4:**
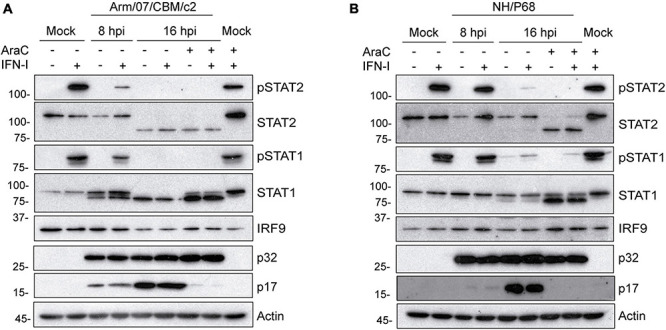
Arm/07/CBM/c2 and NH/P68 interfere with IFN-I signaling by targeting STAT1 and STAT2. PAMs were mock infected or infected with either Arm/07/CBM/c2 **(A)** or NH/P68 strain **(B)** (2 PFU/cell) for 8 or 16 h, in absence or in presence of AraC (40 μg/ml). At 7 or 15 hpi, PAMs were stimulated or not with universal type I IFN (1,000 U/ml). After 1 h of stimulation, cells were collected and lysed in RIPA buffer for further analysis by Western blot. Antibodies against pSTAT2, STAT2, pSTAT1, STAT1, IRF9, early viral protein p32, and actin were employed. As a control of AraC effect on viral DNA replication, a specific antibody against the late viral p17 protein was used, which is only expressed in the absence of AraC.

Next, we analyzed the total expression levels of STAT1 and STAT2 in mock or ASFV infected PAM that were either mock treated or treated with IFN-I at 7 or 15 h post-infection. Importantly, to determine the role of viral replication in the control of STAT expression, infections were performed either in the presence or in the absence of the DNA replication inhibitor cytosine arabinoside (AraC), which is very well known to block the viral cycle prior to viral DNA replication, and to prevent late ASFV genes expression (Rodríguez and Salas, 2013). Interestingly, under these experimental conditions, we found that STAT2 levels were drastically affected during Arm/07/CBM/c2 infection at 16 hpi. In fact, the 113 kDa band corresponding to full length STAT2 was dramatically reduced in the infected cells, while a band of approximately 80 kDa was induced (Figure 4A). These data suggest that infection with the ASFV virulent strain triggers cleavage and/or degradation of STAT2. Importantly, this effect was independent of IFN-I treatment and took place also in the presence of the replication inhibitor AraC. Therefore, it is likely that observed STAT2 degradation is induced at very early times of the infection, prior to viral DNA replication. This is an important observation, and it implies that ASFV has evolved mechanisms to control the IFN-I response that are acting early in the viral life cycle to allow efficient viral replication. Noticeably, NH/P68 infection was also able to impair STAT2 expression through a similar mechanism. Indeed, a decrease in the expression of full length STAT2 and the appearance of the lower molecular weight band were also detected at 16 hpi. However, in this case treatment with AraC was needed to enhance STAT2 degradation (Figure 4B). This is consistent with the involvement of a specific viral protein that is transiently expressed at early time after infection in the control of STAT2 expression. To our surprise, a lower molecular weight STAT1 band was also detected during infection with both Arm/07/CBM/c2 and NH/P68 ASFV strains (Figures 4A,B). This lower band of approximately 80 kDa was detected as early as 8 hpi upon infection with the virulent Arm/07/CBM/c2 strain, and at 16 hpi in cells infected with both strains. This STAT1 lower band could either represent a degradation product, or it may correspond to the β isoform of the STAT1 protein (Schindler et al., 1992). Similar to what we observed for STAT2, this STAT1 modification was enhanced in the presence of AraC, suggesting that early steps of the viral cycle are also implicated in this process. An enhancement of STAT1 expression was detected in the presence of AraC in mock infected cells, which is, nonetheless, counteracted by both ASFV strains. Notably, IRF9 expression was not affected by infection with either virulent or attenuated ASFV strains, supporting the idea that the virus specifically induces STAT1 and STAT2 degradation to counteract IFN-I signaling.

### NH/P68 and Arm/07/CBM/c2 Decrease STAT2 by Proteasomal Degradation

**FIGURE 5:**
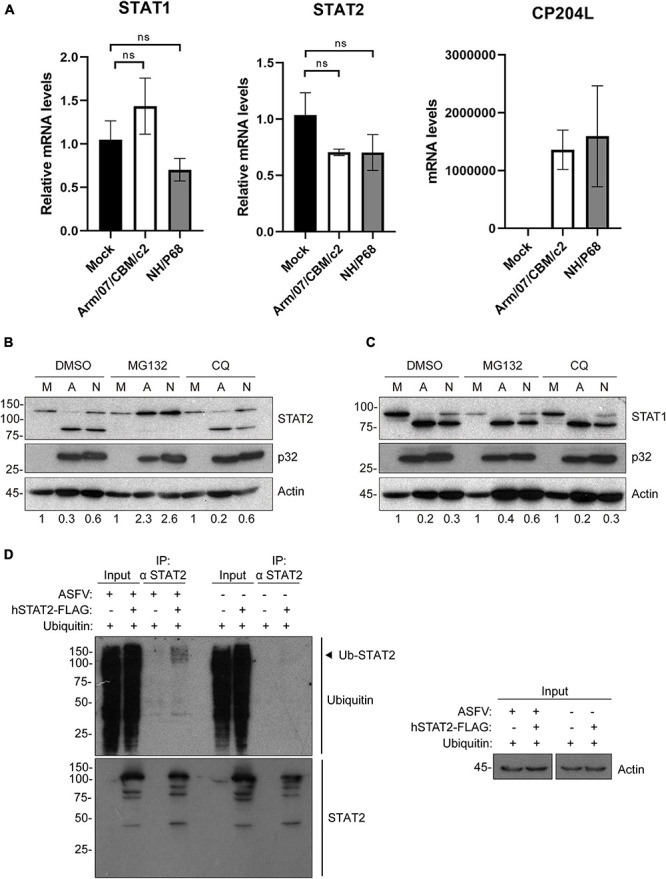
Arm/07/CBM/c2 and NH/P68 infection triggers proteasomal-dependent STAT2 degradation. STAT1 and STAT2 mRNA detection by qRT-PCR **(A)**. PAMs were mock infected or infected with attenuated NH/P68 or with virulent Arm/07/CBM/c2 ASFV strains (1 PFU/cell). Cells were collected at 3 hpi for a qRT-PCR analysis of STAT1 and STAT2 mRNA levels. As a control of infection, viral p32 mRNA (CP204L) was measured. STAT2 **(B)** and STAT1 levels **(C)** were analyzed in presence of the proteasome inhibitor MG132 or the lysosome/autophagosome inhibitor chloroquine (CQ) by Western blot analysis. PAMs were mock infected (M) or infected with Arm/07/CBM/c2 (A) or NH/P68 (N) ASFV strains (3 PFU/cell) and treated with MG132 (20 μM) or chloroquine (50 μM) at 12 hpi. At 16 hpi, cells were collected and lysed for the Western blot analysis. The Western blot bands corresponding to STAT1 (91k Da) and STAT2 (113 kDa) were quantified according with their actin levels and relativized with the corresponding mock control using ImageJ. Immunoprecipitation of STAT2 in mock-infected or infected COS-1 cells **(D)**. COS-1 cells were co-transfected either with pCI-His-hUbiquitin vector and pCAGGS empty vector or hSTAT2-FLAG vector. At 6 h post-transfection cells were infected with Arm/07/CBM/c2 strain (2 PFU/cell). Cells were collected at 16 hpi and lysed for STAT2 immunoprecipitation assay with A/G magnetic beads. Western blot labeling with anti-ubiquitin, anti-STAT2 and anti-actin antibodies is shown. Ubiquitinated STAT2 is indicated with an arrowhead in the figure.

To determine the mechanisms by which STAT1 and STAT2 levels decrease during infection, we then quantified the amount of STAT1 and STAT2 mRNA in mock infected and ASFV infected PAMs. Given that the above results showed a decrease in STAT1 and STAT2 at very early stages of infection, before viral replication, STAT1 and STAT2 mRNAs were measured at early times by qRT-PCR. Expression of the viral gene CP204L was measured as a control. As shown in Figure 5A, we could not detect significant differences in STAT1 and STAT2 mRNA levels between mock infected and NH/P68 or Arm/07/CBM/c2 infected PAM at 3 hpi. This suggests that the observed reduction in their expression levels might occur as a consequence of protein degradation. To identify the protein degradation pathway involved in this process, infected PAMs were treated either with the proteasome inhibitor MG132 or with the lysosome/autophagosome inhibitor chloroquine (CQ), before evaluating the STAT1/2 protein levels by Western blot. As shown in Figure 5B, the levels of STAT2 were fully recovered in infected cells treated with MG132, but not when cells were treated with the lysosomal/autophagosome activity inhibitor. Besides, a dose dependent assay by using increasing concentrations of MG132 inhibitor, confirmed the recovery of STAT2 113 kDa band (Supplementary Figure 4). In contrast, STAT1 expression was not restored in the presence of the inhibitors (Figure 5C), implicating a different mechanism in the control of STAT1 vs. STAT2 during ASFV infection. To further corroborate that STAT2 degradation is mediated by the proteasomal pathway, its ubiquitination status was analyzed by immunoprecipitation (Figure 5D). The Western blot showed that STAT2 is ubiquitinated in infected cells, thus strongly suggesting that ASFV infection triggers proteasomal-mediated STAT2 degradation.

### STAT1 Is Cleaved by Caspase-3 Activation Induced During Arm/07/CBM/c2 and NH/P68 Infection

**FIGURE 6:**
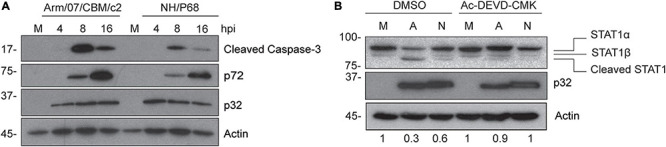
STAT1 is cleaved during caspase-3 activation induced by Arm/07/CBM/c2 and NH/P68 infection. PAM cells were mock infected (M) or infected with Arm/07/CBM/c2 (Arm) or NH/P68 ASFV strains (3 PFU/cell). Cells were collected at 4, 8, and 16 hpi, lysed in RIPA buffer and analyzed by Western blot **(A)**. Antibodies against cleaved caspase-3, early viral protein p32, actin and pig serum 1262 to detect late p72 viral protein were used. PAMs were mock infected (M) or infected with Arm/07/CBM/c2 (A) or NH/P68 (N) ASFV strains (3 PFU/cell) in absence or in presence of the caspase-3 inhibitor Ac-DEVD-CMK (80 μM). At 16 hpi, cells were collected and lysed for the Western blot analysis **(B)**. Antibodies against STAT1, early viral protein p32 and actin were used. The Western blot bands corresponding to STAT1α (91 kDa) were quantified according with their actin levels and relativized with the corresponding mock control using ImageJ. STAT1α (91 kDa), STAT1β (84 kDa) and cleaved STAT1 (80 kDa) bands are highlighted in the figure.

Since caspase-3 is responsible for the inactivation of STAT1 in some cells (King and Goodbourn, 1998), and due to the fact that previous data from our laboratory demonstrated that ASFV infection induces caspase-3 activation and apoptosis early after infection (Carrascosa et al., 2002), we wondered whether the triggering of caspase-3 activation during Arm/07/CBM/c2 and NH/P68 infection is involved in the process of STAT1 degradation. To answer this question, we analyzed the induction of active caspase-3 (17 kDa fragment), in Arm/07/CBM/c2 or NH/P68 infected PAMs at 4, 8, and 16 hpi by Western blot. As shown in Figure 6A, both ASFV strains induced the cleavage of caspase-3 into the active 17 kDa fragment. Remarkably, the caspase-3 activation was less accentuated during the infection with the attenuated NH/P68 isolate with respect to the virulent Arm/07/CBM/c2 strain. As expected, no caspase-3 cleavage was found in mock infected cells. Next, in order to investigate whether STAT1 degradation depends on the caspase-3 enzymatic activity, we monitored STAT1 levels in the absence or in the presence of the caspase-3 inhibitor Ac-DEVD-CMK in mock infected, NH/P68 or Arm/07/CBM/c2 infected cells at 16 hpi. As shown in Figure 6B, STAT1 was strongly degraded during the infection with Arm/07/CBM/c2 at 16 hpi (91 kDa band), while this degradation was less evident with the attenuated isolate NH/P68. Besides, a lower band of approximately 80 kDa was observed, being more evident during the virulent infection. It has been described that STAT1 exists as two isoforms, the full-length STAT1α (91 kDa) and the truncated STAT1β (84 kDa) (Schindler et al., 1992). However, the electrophoretic mobility of the lower band that we observed during infection differs from the STAT1β isoform, suggesting a cleavage product. Importantly, the band corresponding to STAT1α was restored in the presence of caspase-3 inhibitor, while the 80 kDa STAT1 cleaved band was reduced during the treatment. In addition, we found that increasing concentrations of the inhibitor proportionally rescued STAT1α levels and reduced the cleaved band, as observed in the dose-dependent experiment (Supplementary Figure 5). As expected, treatment with the caspase-3 inhibitor did not affect STAT1 levels in the mock infected samples. These data suggest that the degradation of STAT1 observed during both ASFV infections occurs mainly by caspase-3 induced-cleavage.

## Discussion

African swine fever virus (ASFV) is currently the most devastating virus for the pig industry worldwide due to its high virulence and ability to spread. Despite the intensive research effort, the molecular mechanisms responsible for the differences in virulence among the circulating ASFV strains are still far from being understood, and more research in this field is needed to help with the development of efficacious vaccines and antivirals. Pathogenic viruses have developed multiple mechanisms to counteract IFN-I production and/or signaling and dumpen the innate antiviral response (Randall and Goodbourn, 2008). ASFV virulence has also been associated, at least in part, with the ability of the virus to counteract the innate immune response. Indeed, we and others have shown that while virulent ASFV strains are able to inhibit IFN-I production, attenuated isolates are not (Afonso et al., 2004; Gil et al., 2008; Reis et al., 2016). Specifically, we recently reported that the virulent circulating strain Arm/07, and not the attenuated strain NH/P68, impairs IFN-β production by inhibiting the cGAS-STING pathway in PAM (García-Belmonte et al., 2019).

In this study, we further analyzed the ability of ASFV to interfere with the host antiviral response by counteracting the JAK/STAT signaling pathway. Our results demonstrate for the first time that both attenuated and virulent ASFV strains are able to counteract the IFN-I signaling pathway. Interestingly, this is likely done by inducing STAT1 and STAT2 degradation through two independent mechanisms. We show that ASFV infection triggers STAT2 degradation via the proteasome as well as STAT1 cleavage by activation of caspase-3. The loss of STAT1 and STAT2 function observed in our study is consistent with the degradation of these cellular proteins, further demonstrating that IFN-dependent phosphorylation of both STAT1 and STAT2 is impaired in infected cells and that the ISGF3 complex is not detected in the nucleus and does not trigger ISGs expression. In this regard, Vaccinia virus, a large DNA virus which shares several features with ASFV, employs the proteasomal degradation to activate the VH1 phosphatase, which is required to dephosphorylate STAT1 and prevents the IFN I-mediated antiviral response (Schmidt et al., 2013). However, *Portugal et al.* claimed that neither virulent L60 nor attenuated NH/P68 displayed any effect on IFN-I dependent genes such as ISG15 or PKR whereas the virulent strain affected to MxA expression and showed that phosphorylation of STAT1/2 was not affected during ASFV infection (Portugal et al., 2018). This discrepancy might reflect the use of different experimental systems.

Importantly, in agreement with previous data showing that virulent ASFV isolates are more efficient at inhibiting the host antiviral response (Gil et al., 2003, 2008; Fishbourne et al., 2013; Reis et al., 2016; García-Belmonte et al., 2019), we observed a more dramatic degradation of STAT1 and STAT2 in cells infected with the Arm/07/CBM/c2 isolate. Whether these differences are due to the expression of different IFN antagonists by the two viruses, or to variations in the same viral gene/s remains to be determined, and is an interesting topic for future investigation. Nevertheless, the fact that both viruses are capable to counteract the JAK/STAT signaling pathway suggests that hampering innate immune activation is essential to allow viral cycle achievement. In addition, this would help explain why the attenuated isolate, despite not being able to inhibit IFN-I production (García-Belmonte et al., 2019), can still efficiently replicate both in pigs and in PAM (Sánchez et al., 2017).

Several viruses are known to trigger STAT1 and STAT2 degradation to counteract the antiviral response induced by IFN-I. Proteasome-dependent degradation of STAT2 has been detected during infection with Zika (Grant et al., 2016), Dengue (Ashour et al., 2009), Human Cytomegalovirus (HCMV) (Le-Trilling et al., 2020), Respiratory syncytial virus (Elliott et al., 2007) and PRRSV (Yang et al., 2019). Such degradation is usually mediated by the direct interaction of a specific viral protein with host E3 ubiquitin ligases that are hijacked during infection (Morrison et al., 2013; Le-Trilling et al., 2020). Our data reveal a decrease in STAT2 levels (113 kDa band) that is accompanied by the appearance of a lower molecular weight band, which is negatively affected in the presence of the inhibitor MG132. In this regard, it is noteworthy the fact of two models of protein selection for proteasome degradation (reviewed in Belizario et al., 2008). While one model states that the attachment of ubiquitin chains serves as a recognition element to 19S complexes and its consequent unfolding, to get access to the 20S catalytic complex (Glickman and Ciechanover, 2002), the second model includes a previous cleavage of the protein at the PEST motif promoting its access into the 26S/20S proteasome. In addition, there is evidence of an accelerated and more efficient degradation by the 20S proteasome that does not require poly-ubiquitination chains if an unstructured region is recognized (Liu et al., 2003; Prakash et al., 2004). Having this knowledge in mind, and in the light of our results, while we could confirm that the virus-induced degradation of STAT2 depends on the proteasomal degradation complex, we may also hypothesize that the observed lower band could be a product of this alternative proteolytic process induced by the virus, to further destabilize and accelerate its proteasomal degradation.

In our study, although we did not identify the viral factor/s that are direct responsible for the observed STAT2 degradation, we convincingly showed that treatment with the viral DNA replication inhibitor AraC significantly enhances this degradation, implicating an early viral product that may accumulate to higher levels when DNA replication is inhibited. Interestingly, ASFV encodes for a putative E2 ubiquitin conjugating enzyme (ORF I215L) that is expressed both at early and late time points after infection, and has sequence homology with eukaryotic E2-ubiquitin like enzymes (Freitas et al., 2018). In addition, expression of the MGF-505-7R gene, has been recently associated with the inhibition of type I and type II IFN signaling (Correia et al., 2013). Studies are currently ongoing to address the involvement of these viral proteins in the degradation of STAT2.

Remarkably, we also show that infection with both virulent and attenuated ASFV strains results in impaired expression of full-length STAT1. Specifically, a 80 kDa faster migrating form of STAT1, which differs from the STAT1β isoform (84 kDa), was detected by Western blot during infection with NH/P68 and Arm/07/CBM/c2. However, unlike STAT2, STAT1 expression could not be rescued by treatment with proteasome or lysosomal/autophagosomal inhibitors. It was previously described by our group and by others, that caspase-3 and caspase-9 are induced early upon infection, and that expression of anti- and pro-apoptotic genes is tightly regulated throughout the ASFV life cycle (Chacún et al., 1995; Revilla et al., 1997; Brun et al., 1998; Nogal et al., 2001; Afonso et al., 2004; Hernáez et al., 2004). In addition, STAT1 was identified as a caspase-3 target in the context of apoptosis (King and Goodbourn, 1998; Adrain and Martin, 2001; Slee et al., 2001; Licht et al., 2014). The 81 kDa cleaved form of human STAT1 induced during apoptosis described in *King et al*., matches with the lower STAT1 band observed in our experiments during ASFV infection. This 81 kDa band is distinguishable from the STAT1β isoform whose weight corresponds to 84 kDa band that migrates with slightly slower mobility than the cleaved band. *King et al*. identified the aspartic acid residue 694 as a cutting residue of caspase-3, which, interestingly, is conserved in porcine STAT1. In addition, we confirmed that caspase-3 is activated during NH/P68 and Arm/07/CBM/c2 infections in PAM being more pronounced during infection with the virulent strain, which correlated with a more effective degradation of STAT1 with this isolate. Strikingly, treatment with the caspase-3 inhibitor Ac-DEVD-CMK restored the levels of full length STAT1 and decreased the 80 kDa lower band, clearly indicating that caspase-3 and early induction of apoptosis are required for STAT1 cleavage during ASFV infection.

In summary, in this study we have demonstrated that both virulent Arm/07/CBM/c2 and attenuated NH/P68 strains display different mechanisms to counteract the JAK/STAT pathway, highlighting the importance of the control of this pathway for ASFV innate immune evasion. Furthermore, the differences on the efficiency of inhibition of IFN-I signaling observed during infection with the virulent and the attenuated ASFV strain suggest that the molecular mechanism for ASFV virulence may rise from different levels of control of this pathway. The identification of specific ASFV proteins and their mechanisms counteracting the JAK/STAT pathway are currently under study.

## Data Availability Statement

The raw data supporting the conclusions of this article will be made available by the authors, without undue reservation.

## Author Contributions

YR and DP-N conceived and design of the study. ER wrote the first draft of the manuscript and performed most of the experiments. RG-B contributed to the experiments performance, methodology, and to the formal analysis. AG-S, LM, DP-N, and YR contributed to the investigation, supervision, visualization, and to the review/editing of the manuscript. All authors have read and agreed to the published version of the manuscript.

## Conflict of Interest

The authors declare that the research was conducted in the absence of any commercial or financial relationships that could be construed as a potential conflict of interest.

## Publisher’s Note

All claims expressed in this article are solely those of the authors and do not necessarily represent those of their affiliated organizations, or those of the publisher, the editors and the reviewers. Any product that may be evaluated in this article, or claim that may be made by its manufacturer, is not guaranteed or endorsed by the publisher.

## Supplementary Material

Supplementary Figure 1 NH/P68 and Arm/07/CBM/c2 infection impair IFN-I induced-ISG15 expression. Western blot analysis of unconjugated ISG15 (15 kDa) and conjugated ISG15 (35 kDa) in mock-infected and infected COS-1 cells treated or not with IFN-I. COS-1 cells were mock-infected or infected with either NH/P68 or Arm/07/CBM/c2 strains (1 PFU/cell, 16 hpi) and treated or not with type I IFN (1,000 U/ml) at 15 hpi, during 1 hour. Antibodies against ISG15 and actin were used.

Supplementary Figure 2 NH/P68 and Arm/07/CBM/c2 strains counteract IRF9/STAT1/STAT2 nuclear translocation at late times of infection. PAMs were mock infected or infected with NH/P68 or Armenia/07/CBM/c2 (Arm) (1 PFU/cell). At 7 or 15 hpi, cells were untreated or treated with universal type I IFN (250 U/ml). After 1 h of treatment, cells were fixed and stained with DAPI (blue), anti-p32 (red) and anti-IRF9, anti-STAT1 or anti-STAT2 (green) antibodies and examined by a confocal microscope. Individual channel images are shown.

Supplementary Figure 3 NH/P68 and Arm/07/CBM/c2 strains inhibits STAT1 and STAT2 phosphorylation at late times of infection. PAMs were mock infected or infected with NH/P68 or Armenia/07/CBM/c2 (Arm) (1 PFU/cell). At 7 or 15 hpi, cells were untreated or treated with universal type I IFN (250 U/ml). After 1 h of treatment, cells were fixed and stained with DAPI (blue), anti-p32 (red), and anti-pSTAT1 or anti-pSTAT2 (green) antibodies and examined by a confocal microscope. Individual channel images are shown.

Supplementary Figure 4 ASFV-induced STAT2 degradation depends on the proteasomal degradation pathway. PAMs were mock infected or infected with attenuated NH/P68 ASFV strain (2 PFU/cell) and treated or not with increasing concentrations of MG132 (1, 5, or 20 μM) at 12 hpi. At 16 hpi, cells were collected and lysed for the Western blot analysis. Antibodies against STAT2, viral p32 protein and actin were used.

Supplementary Figure 5 STAT1 cleavage is induced by caspase-3 during ASFV infection. PAMs were mock infected or infected with attenuated NH/P68 ASFV strain (2 PFU/cell) in absence or in presence of increasing concentrations of caspase-3 inhibitor Ac-DEVD-CMK (10, 40, or 100 μM). At 16 hpi, cells were collected and lysed for the Western blot analysis. Antibodies against STAT1, early viral protein p32 and actin were employed. The Western blot bands corresponding to STAT1α (91 kDa), STAT1β (84 kDa), and cleaved STAT1 (81kDa) are indicated in the figure.
