# Supplementary material for: African Swine Fever Virus Induces STAT1 and STAT2 Degradation to Counteract IFN-I Signaling
Source: Front Microbiol. 2021 Aug 26;12:722952. doi: 10.3389/fmicb.2021.722952 (PMC8427279; doi:10.3389/fmicb.2021.722952)
Supplement: Supplementary file 6 [file Data_Sheet_1.docx]

**Supplementary Figure 1. NH/P68 and Arm/07/CBM/c2 infection impair IFN-I induced-ISG15 expression.** Western blot analysis of unconjugated ISG15 (15 kDa) and conjugated ISG15 (35 kDa) in mock-infected and infected COS-1 cells treated or not with IFN-I. COS-1 cells were mock-infected or infected with either NH/P68 or Arm/07/CBM/c2 strains (1 PFU/cell, 16hpi) and treated or not with type I IFN (1,000 U/ml) at 15 hpi, during 1 hour. Antibodies against ISG15 and actin were used.

**Supplementary Figure 2.** **NH/P68 and Arm/07/CBM/c2 strains counteract IRF9/STAT1/STAT2 nuclear translocation at late times of infection.** PAMs were mock infected or infected with NH/P68 or Armenia/07/CBM/c2 (Arm) (1 PFU/cell). At 7 or 15 hpi, cells were untreated or treated with universal type I IFN (250 U/ml). After 1 hour of treatment, cells were fixed and stained with DAPI (blue), anti-p32 (red) and anti-IRF9, anti-STAT1 or anti-STAT2 (green) antibodies and examined by a confocal microscope. Individual channel images are shown.

**Supplementary Figure 3.** **NH/P68 and Arm/07/CBM/c2 strains inhibits STAT1 and STAT2 phosphorylation at late times of infection.** PAMs were mock infected or infected with NH/P68 or Armenia/07/CBM/c2 (Arm) (1 PFU/cell). At 7 or 15 hpi, cells were untreated or treated with universal type I IFN (250 U/ml). After 1 hour of treatment, cells were fixed and stained with DAPI (blue), anti-p32 (red) and anti-pSTAT1 or anti-pSTAT2 (green) antibodies and examined by a confocal microscope. Individual channel images are shown.

**Supplementary Figure 4. ASFV-induced STAT2 degradation depends on the proteasomal degradation pathway.** PAMs were mock infected or infected with attenuated NH/P68 ASFV strain (2 PFU/cell) and treated or not with increasing concentrations of MG132 (1, 5 or 20 µM) at 12 hpi. At 16 hpi, cells were collected and lysed for the Western blot analysis. Antibodies against STAT2, viral p32 protein and actin were used.

**Supplementary Figure 5. STAT1 cleavage is induced by caspase-3 during ASFV infection.** PAMs were mock infected or infected with attenuated NH/P68 ASFV strain (2 PFU/cell) in absence or in presence of increasing concentrations of caspase-3 inhibitor Ac-DEVD-CMK (10, 40 or 100 µM). At 16 hpi, cells were collected and lysed for the Western blot analysis. Antibodies against STAT1, early viral protein p32 and actin were employed. The Western blot bands corresponding to STAT1α (91 kDa), STAT1β (84 kDa) and cleaved STAT1 (81kDa) are indicated in the figure.
